# Supplementary material for: Complexome profiling of the Chlamydomonas psb28 mutant reveals TEF5 as an early PSII assembly factor
Source: Plant Cell. 2025 Mar 17;37(6):koaf055. doi: 10.1093/plcell/koaf055 (PMC12164590; doi:10.1093/plcell/koaf055)
Supplement: koaf055_Supplementary_Data [file koaf055_supplementary_data.zip › psb28_interactive.zip!/plots/intensity/qualityPlots/peptides/LHCBM5_Cre03.g156900.t1.2.html]

### Cre03.g156900.t1.2

Trivial name: LHCBM5  
SeqCoverage [median]: 53.00  
Mass: 28.69 kDa  
  
 

| Sequence | MissedCleavage | Mass [Da] | wtIntensity | muIntensity | Score |
| --- | --- | --- | --- | --- | --- |
| AGWWSNGGNEK | 0 | 1204.53 | 2053.67 | 12234.46 | 160.53 |
| EKAGWWSNGGNEK | 1 | 1461.66 | 0.00 | 954.00 | 62.29 |
| GPLANLDEHLASPFTSNAFTYAQK | 0 | 2591.27 | 187256.84 | 243584.18 | 252.47 |
| KAAPASAQK | 1 | 870.49 | 871.36 | 2774.63 | 136.21 |
| LHPGGQFFDPLGLAEDPDAFAELK | 0 | 2583.26 | 404086.15 | 469564.30 | 154.86 |
| LHPGGQFFDPLGLAEDPDAFAELKVK | 1 | 2810.43 | 14436.37 | 15285.15 | 111.40 |
| LSAFYGPDR | 0 | 1024.50 | 1108516.14 | 1435940.64 | 219.25 |
| NGTPIVEPVWFK | 0 | 1385.73 | 24978.74 | 0.00 | 147.78 |
| TVVNVQAK | 0 | 857.50 | 10044.11 | 15285.45 | 113.89 |
| VNGGPAGEGLDK | 0 | 1112.55 | 212360.75 | 341950.19 | 256.96 |
| VNGGPAGEGLDKLHPGGQFFDPLGLAEDPDAFAELK | 1 | 3677.80 | 37005.17 | 0.00 | 80.24 |
|  |  |  | 2001609.3 | 2537573.0 |  |
